# Antimicrobial-Resistant *Escherichia coli* Distribution and Whole-Genome Analysis of Sequence Type 131 *Escherichia coli* Isolates in Public Restrooms in Taiwan

**DOI:** 10.3389/fmicb.2022.864209

**Published:** 2022-04-13

**Authors:** Szu-Min Chang, Jenn-Wei Chen, Chin-Shiang Tsai, Wen-Chien Ko, Joy Scaria, Jiun-Ling Wang

**Affiliations:** ^1^Institute of Basic Medical Sciences, College of Medicine, National Cheng Kung University, Tainan, Taiwan; ^2^Department of Microbiology and Immunology, College of Medicine, National Cheng Kung University, Tainan, Taiwan; ^3^Department of Internal Medicine, National Cheng Kung University Hospital, Dou-Liou Branch, College of Medicine, National Cheng Kung University, Yunlin, Taiwan; ^4^Institute of Clinical Medicine, College of Medicine, National Cheng Kung University, Tainan, Taiwan; ^5^Department of Medicine, College of Medicine, National Cheng Kung University, Tainan, Taiwan; ^6^Department of Internal Medicine, National Cheng Kung University Hospital, College of Medicine, National Cheng Kung University, Tainan, Taiwan; ^7^Department of Veterinary and Biomedical Sciences, South Dakota State University, Brookings, SD, United States

**Keywords:** extended-spectrum beta-lactamases, sequence type 131, *E. coli*, whole-genome analysis, restrooms, fimH, fimbriae gene

## Abstract

The threat of antibiotic-resistant bacteria to public health may originate from public restrooms. To better understand the community burden of antimicrobial-resistant *Escherichia coli* and sequence type complex 131 *E. coli* (STc131) in the public restroom, we performed a surveillance in public restrooms in southern Taiwan. Swabs were sampled from randomly selected public restrooms in Tainan, Taiwan in 2019. Antimicrobial susceptibility, phylogenetic grouping, and multiplex PCR were performed for the major ST complex in the B2 phylogenetic group. If STc131 isolates were identified, the whole-genome sequencing was performed. A total of 613 collection sites found 132 sites (21.5%) positive for *E. coli*. The most common phylogenetic group was A (30.9%) followed by B2 (30.3%). Ceftriaxone-resistant *E. coli* and extended-spectrum β-lactamases–producing *E. coli* were found in 2.4 and 1.0% of total public restrooms, respectively. The isolates in rural areas had higher ceftriaxone non-susceptibility than those in the city centers (3.9 vs. 1.2%, *P* = 0.038). Nine STc131 isolates were found in public restrooms, and most (77.8%) belonged to the subtype fimH41, whereas 22.2% belonged to fimH30. With the inclusion of STc131 isolates from human and dog fecal colonization in Taiwan, whole-genome sequencing was performed in 35 isolates. A large cluster of fimH41 in SNP-tree and GrapeTree was found from different sources (human, dog, and environment) and geographical areas. In conclusion, our surveillance of antimicrobial-resistant *E. coli* showed a higher prevalence of *E. coli* detected in public restrooms in the rural areas compared to those in city centers. The whole-genome sequence implies that fimH41 STc131 strains are successfully circulated in the community in Taiwan.

## Introduction

In recent years, extended-spectrum beta-lactamases (ESBL)–producing or ceftriaxone-resistant *Escherichia coli* infections have rapidly increased in both community and healthcare-associated settings, leading to a serious threat to global public health (Manges et al., 2019). The epidemic clone of ST131 and increases in antimicrobial use in the community may explain this phenomenon (Mathers et al., 2015; MacFadden et al., 2019; Whitmer et al., 2019; Castanheira et al., 2021). In addition, more studies on the detection of ESBL-producing *E. coli* in asymptomatic fecal carriers and fecal waste streams have recently been reported (Woerther et al., 2013; Bezabih et al., 2021). Southeast Asia and Africa have the highest burden of defecated feces containing ESBL-producing *E. coli* (Berendes et al., 2020).

A previous study from the United Kingdom showed a commonality between bacteremic ESBL-producing *E. coli* isolates and those from feces and sewage (Day et al., 2019). The human-to-human oral–fecal route is the most frequent route of transmission for human-adapted ESBL-producing *E. coli* (Day et al., 2019; Berendes et al., 2020). In a study with environmental surveillance in nursing homes in the Netherlands, ESBL-producing *E. coli* were most frequently identified in the toilets (Overdevest et al., 2016). *E. coli* isolates are commonly isolated in public toilets and mostly occur in airports, bus terminals, and universities (Fankem et al., 2006). ESBL-producing *E. coli* in the sludge may contaminate surface water and groundwater, leading to community spread (Hossain et al., 2021).

A study in Germany implied that transmission from person-to-person *via* contact surfaces may occur both in public toilets and in households (Gerhardts et al., 2012). The transmission model by simulating the chain of infection from stool showed *E. coli* can be transferred *via* the toilet brush and the door handle to the gloved hand (Gerhardts et al., 2012). In addition, a study of public restrooms in Taiwan showed areas near toilet bowls, squat toilets, and urinals were highly contaminated (Lee and Tham, 2021). Bacteria of potential fecal origin may contaminate human hands, and hand washing can reduce the presence of bacteria (Judah et al., 2010; Burton et al., 2011). Therefore, the threat of antibiotic-resistance genes to public health may originate from public restrooms and sewage (Mkrtchyan et al., 2013; Pepper et al., 2018; Hendriksen et al., 2019). Few studies have surveyed public restrooms for environmental ESBL-producing *E. coli* (Mohamed et al., 2015). To better understand the burden of ESBL-producing or ceftriaxone-resistant *E. coli* and ST131 *E. coli* in public restrooms, we performed surveillance in public restrooms in southern Taiwan. Because whole-genome sequences help to track the spread of individual strains (Robins-Browne et al., 2016; Yehouenou et al., 2021), we performed genomic epidemiological investigations of ST131 *E. coli* isolates to compare the environmental isolates with human isolates.

## Materials and Methods

### Sampling Method

According to the guideline issued by the Environmental Protection Admiration in Taiwan, the recommended frequency of cleaning of public restrooms should be at least twice a day. If the environment is obviously contaminated, then cleaning of public restrooms must be performed more frequently, even as frequently as every two hours, if necessary. Soap or detergent was used for general surface cleaning. Diluted bleach was used for disinfecting of sink U-trap, urinal, and toilet bowl.

Dry sterile cotton swabs (BD™ transport tubes) were used to collect samples from randomly selected public restrooms in Tainan, Taiwan, *via* convenience sampling from 1 March to 31 May 2019. In each public restroom, we collected one swab from the toilet bowl or rim and one swab from the wash basin. Finally, there were 613 sites including 312 from toilet bowls or rims and 301 from the washbasins. There were 302 samples from male restrooms, 301 samples from female restrooms, and 10 samples from unisex restrooms.

The swab area was approximately 100 cm^2^, according to a previous guideline in environmental sampling (Chai et al., 2018). All the specimens were transferred to the laboratory immediately after sample collection at room temperature.

We recorded the basic data of facilities, including male, female, and unisex toilets. The locations of public restrooms in this study included rural areas (*n* = 283), city centers (*n* = 330), and buildings [department stores (*n* = 32) and 4/5-star hotels (*n* = 28), public parks (*n* = 81), gas stations (*n* = 105), freeway service areas(*n* = 20), transportation hubs (*n* = 54), parking lots (*n* = 8), tourist destinations (*n* = 166), shopping malls (*n* = 39), universities (*n* = 28), and hospitals (*n* = 52)]. The swab samples were directly inoculated on a selective agar medium CHROMagar™ ECC (CHROMAgar ^®^, France) for screening of *E. coli*, and up to two *E. coli* colonies were selected per sample. We used conventional tube biochemical testing for confirmation the isolates as *E. coli* (Garcia, 2010).

### Antimicrobial-Resistant Test and Phylogenetic Group Study for *Escherichia coli* Isolates

Among the 613 collection sites, 132 sites (21.5%) tested positive for *E. coli* including 20 sites had two colonies of *E. coli* found. The disk diffusion method for antibiotic susceptibility was performed in these 152 isolates for cefazolin, amoxicillin, ciprofloxacin, ceftriaxone, ceftazidime, gentamicin, imipenem, amikacin, and trimethoprim/sulfamethoxazole (TMP/SMX), according to the Clinical and Laboratory Standards Institute (Cockerill and Clinical and Laboratory Standards Institute, 2011). The ESBL phenotypic confirmatory disk diffusion test was performed with cefotaxime (30 μg) and ceftazidime (30 μg) disks, individually and in combination with clavulanic acid (10 μg), following the published guidelines (Watt et al., 2000; Cockerill and Clinical and Laboratory Standards Institute, 2011). We also performed ESBL gene screening including *bla*CTX-M, *bla*SHV, *bla*TEM, and *bla*OXA-1 by PCR in ESBL-producing *E. coli* isolates (Dallenne et al., 2010).

Each *E. coli* isolate (*n* = 152) was subjected to phylogenetic group analysis. DNA extraction was performed with MasterPure™ complete DNA and RNA purification kit (Lucigen Corp., Middleton, WI, United States) following the manufacturer’s instructions. Phylogenetic groups were determined using primers designed by Clermont et al. (2013). An allele-specific PCR method was used to detect the nine main *E. coli* phylogroup B2 lineages (sequence type complex, STc), including STc73, STc12, STc127, STc14, STc95, STc131, STc141, STc144, and STc372 (Clermont et al., 2014).

### Whole-Genome Sequence

We performed whole-genome sequencing on the STc131 isolates found in this study and our previous study, including fecal colonization in asymptomatic humans in Kaohsiung, Taiwan (*n* = 16) (Wu et al., 2019) and anal swabs from companion animals (dogs) (*n* = 10) in Chiayi, Taiwan (Chen et al., 2020). A Nextera XT DNA library preparation kit (Illumina, San Diego, CA, United States) was used to construct a genomic library. The Illumina MiSeq platform with paired-end chemistry was used for the whole-genome sequencing. All contigs were submitted to the Enterobase *E. coli*/Shigella section^1^ (Zhou et al., 2020) and the CGE Finder Series (Centre for Genomic Epidemiology, Technical University of Denmark). FimH typing was performed using the FimTyper (Roer et al., 2017). The Enterobase website generates GrapeTree figures using the neighbor-joining algorithm (Zhou et al., 2018) by clustering based on core genome multilocus sequence typing (cgMLST). Distances between genomes were calculated using the number of shared cgMLST alleles, and genomes were linked using a single-linkage clustering criterion. The acquired genes and chromosomal mutations mediating antimicrobial resistance in STc131 *E. coli* isolates were investigated using ResFinder 4.0 from the CGE Finder Series (Camacho et al., 2009; Zankari et al., 2017; Bortolaia et al., 2020).

### Statistical Analyses

For statistical analyses, we used SPSS version 20.0 for Windows (SPSS Inc., Armonk, NY, United States). Chi-square or Fisher’s exact tests were used to analyze categorical variables. Statistical significance was set at *P* < 0.05.

## Results

### Distribution of *Escherichia coli* in Public Restrooms

**FIGURE 1:**
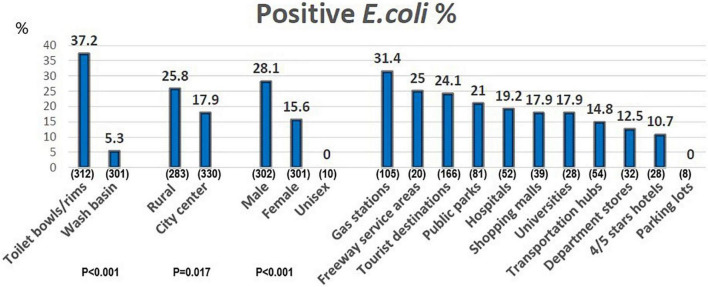
The percentage of positive *E. coli* in different public restrooms. *X*-axis: different public restroom, the numbers in parentheses means the sampling numbers. *Y*-axis: the percentage of positive *E. coli.*

Among the 613 collection sites, 132 sites (21.5%) tested positive for *E. coli* including 20 sites had two colonies of *E. coli* found. The percentage of positive *E. coli* in different public restrooms was shown in Figure 1. Public restrooms located in rural areas were more likely to find *E. coli* (25.8 vs. 17.9%; *P* < 0.05). Of the restrooms, 31.4% in gas stations, 25.0% in freeway service areas, 24.1% of tourist destinations, and 21% of parks could isolate *E. coli*. In contrast, *E. coli* were not detected in restrooms in parking lots; meanwhile, 10.7% were detected in 4/5 stars hotels and 12.5% in department stores (Figure 1). Basic data on public restrooms in rural areas and city centers are shown in Table 1. All public restrooms of department stores and 4/5 stars hotels in this study were located in the city center. The other distributions are listed in Table 1.

**TABLE 1:** Baseline data on sample collection from public restrooms in this study.

	Rural area (*n* = 283)	City center (*n* = 330)	Total (*n* = 613)
	Numbers of sampling (% of rural area)	Numbers of sampling (% of city center)	Numbers of sampling (% of total)
Toilet bowl/rim	145 (51.2)	167 (50.6)	312 (50.9)
Wash basin	138 (48.8)	183 (49.4)	301 (49.1)
**Sex**			
Male	138 (48.4)	164 (49.7)	302 (49.3)
Female	137 (48.8)	164 (49.7)	301 (49.1)
Unclassified	8 (2.8)	2 (0.6)	10 (1.6)
**Restroom category**			
Department store	0	32 (9.7)	32 (5.2)
4/5-star hotel	0	28 (8.5)	28 (4.6)
Public park	42 (14.8)	39 (11.8)	81 (13.2)
Gas station	60 (21.2)	45 (13.6)	105 (17.1)
Freeway service area	16 (5.7)	4 (1.2)	20 (3.3)
Transportation hub	30 (10.6)	24 (7.3)	54 (8.8)
Parking lot	4 (1.4)	4 (1.2)	8 (1.3)
Tourist destination	96 (33.9)	70 (21.2)	166 (27.1)
Shopping mall	7 (2.5)	32 (9.7)	39 (6.4)
University	8 (2.8)	20 (6.1)	28 (4.6)
Hospital	20 (7.1)	32 (9.7)	52 (8.5)

### Antimicrobial-Resistant *Escherichia coli* in Public Restrooms

We further analyzed antimicrobial susceptibility of 152 *E. coli* isolates from the 613 collection sites. Ceftriaxone or ceftazidime non-susceptible *E. coli* were found in 15 isolates (9.8% in all isolates, 2.4% in all public restrooms). In addition, ESBL-producing *E. coli* isolates were found in six isolates (3.9% in all isolates and 1.0% in all public rooms) by ESBL phenotypic testing (Table 2). Imipenem or amikacin resistance was not observed in this study. Comparing geographical data, we found that rural areas had a higher proportion of amoxicillin or ceftriaxone non-susceptibility (*P* < 0.05). Of the six ESBL-producing *E. coli* isolates, ESBL gene can be found in *bla*CTX-M group 1 (*n* = 4), *bla*TEM (*n* = 3), and *bla*CTX-M group 9 (*n* = 2).

**TABLE 2:** Geographical distribution of resistant *E. coli* across 613 public restrooms in rural areas and city centers in Tainan.

	Rural area *N* = 283	City center *N* = 330	Total *N* = 613	*P*- value (Rural area vs. city center)
Numbers of sites with NS isolates found (% of numbers in rural area, city center, and total)				

Cefazolin NS	28 (9.9)	23(7.0)	51 (8.3)	0.240
Ceftriaxone NS	11 (3.9)	4 (1.2)	15 (2.4)	0.038
Ceftazidime NS	11 (3.9)	4 (1.2)	15 (2.4)	0.038
Ciprofloxacin NS	5 (1.8)	3 (0.9)	8 (1.3)	0.481
TMP-SMX NS	28 (9.9)	19 (5.8)	47 (7.7)	0.067
Amoxicillin NS	20 (7.1)	10 (3.0)	30 (4.9)	0.024
Gentamicin NS	4 (1.4)	1 (0.3)	5 (0.8)	0.187
ESBL	5 (1.8)	1 (0.3)	6 (1.0)	0.10
ST131	3 (1.1)	6 (1.8)	9 (1.5)	0.516

*NS: not susceptible including resistant and intermediate.*

*Any collection site with one antimicrobial non-susceptible isolate was calculated. The number of intermediate (I) and resistant (R) in cefazolin: rural area: (15, 13); city center: (17, 6).*

*The number of intermediate (I) and resistant (R) in ceftriaxone: rural area: (2, 9); city center: (2, 2).*

*The number of intermediate (I) and resistant (R) in ceftazidime: rural area: (0, 11); city center: (1, 3).*

*The number of intermediate (I) and resistant (R) in ciprofloxacin: rural area: (2, 3); city center: (1, 2).*

*The number of intermediate (I) and resistant (R) in TMP-SMX: rural area: (0, 28); city center: (1, 18).*

*The number of intermediate (I) and resistant (R) in amoxicillin: rural area: (9, 11); city center: (4, 6).*

*All gentamicin NS isolates were resistant.*

*TMP-SMX, trimethoprim-sulfamethoxazole; ESBL, extended-spectrum β-lactamases; ST131, sequence type 131.*

### Phylogenetic Group and Sequence Type Complex Distributions in Public Restrooms

**FIGURE 2:**
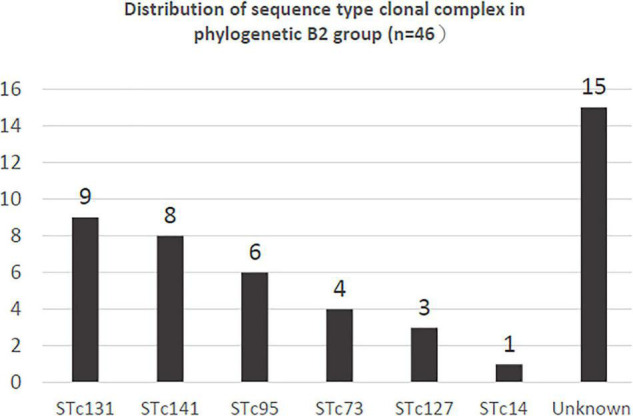
The distribution of sequence type complexes (STc) among the 46 phylogenetic group B2 *E. coli* isolates in this study.

We analyzed the phylogenetic groups in greater detail for total 152 isolates. The most common phylogenetic group was A (30.9%), followed by B2 (30.3%), B1 (10.5%), D (9.9%), C (6.6%), E (5.3%), F (3.9%), and non-groupable (2.6%) (Table 3). The distributions of the phylogenetic group are shown in Table 3. The six ESBL-producing *E. coli* isolates included phylogenetic group A (*n* = 1), B1 (*n* = 1), D (*n* = 1), E (*n* = 2), and F (*n* = 1). Of the 46 isolates of phylogenetic B2 group, the distribution of STs is shown in Figure 2. The most common STc in the B2 group was STc131 (*n* = 9), followed by STc141 (*n* = 8) and STc95 (*n* = 6). Nine isolates belong to STc131 was found, which accounts for 1.5% of all public restrooms and 6.8% of the sites with *E. coli*. STc131 was more often found in the city center than rural area but was not statistically significant (*P* > 0.05) (Table 2). All of nine STc131 isolates were susceptible to ciprofloxacin and ceftriaxone, and five were non-susceptible to cefazolin or TMP/SMX, and only one was non-susceptible to gentamicin.

**TABLE 3:** The distribution of phylogenetic groups in 152 *E. coli* isolates found in public restrooms.

	A	B1	B2	C	D	E	F	Non-groupable	Total
No of isolates	47 (30.9)	16 (10.5)	46 (30.3)	10 (6.6)	15 (9.9)	8 (5.3)	6 (3.9)	4 (2.6)	152
(% of *n*)									

Male	30 (30)	14 (14.0)	25 (25.0)	8 (8)	8 (8)	6 (6)	5 (5)	4 (4)	100

Wash basin	2 (13.3)	3 (20.0)	5 (33.3)	1 (6.7)	1 (6.7)	2 (13.3)	1 (6.7)	0	15
Toilet bowl/rim	45 (32.8)	13 (9.5)	41 (29.9)	9 (6.6)	14 (10.2)	6 (4.4)	5 (3.6)	4 (2.9)	137
Rural area	28 (33.3)	7 (8.3)	21 (25.0)	9 (10.7)	9 (10.7)	5 (6.0)	3 (3.6)	2 (2.4)	84
City center	19 (27.9)	9 (13.2)	25 (36.8)	1 (1.5)	6 (8.8)	3 (4.4)	3 (4.4)	2 (2.9)	68

### Fimbriae Gene Type and Whole-Genome Analysis of Sequence Type Complex 131 *Escherichia coli*

**FIGURE 3:**
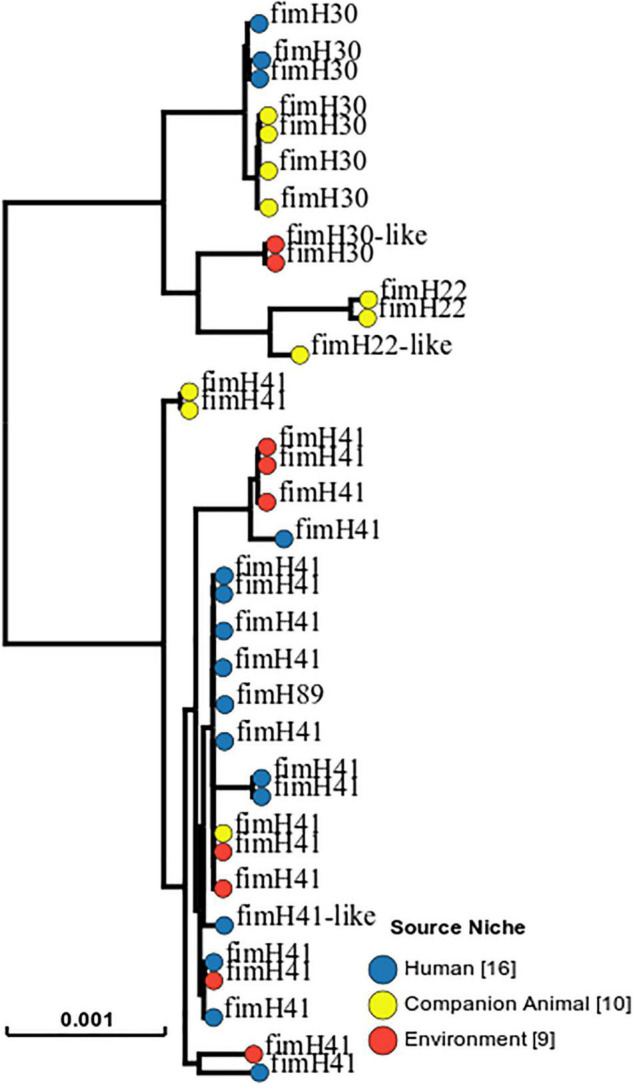
SNP-tree of 35 representative ST131 *E. coli* genomes collected from human feces (*n* = 16), dog feces (*n* = 10), and public restrooms (*n* = 9). One isolate (No. 13) in an asymptomatic fecal colonization of fimH30 was used as a reference.

Whole-genome analysis of 35 *E. coli* isolates of STc131 in southern Taiwan from three sources (public restrooms: *n* = 9; human feces: *n* = 16; dog feces: *n* = 10) showed that 33 isolates were ST131 and two isolates were ST5640. The most common fimH type was fimH41 (62.9%, *n* = 22), followed by fimH30 (25.7%, *n* = 9), fimH22 (4.5%; *n* = 3), and fimH89 (1 SNP to fimH41, 2.8%; *n* = 1). The SNP tree generated by EnteroBase of 16 isolates collected from human feces (Wu et al., 2019), 10 from dog feces (Chen et al., 2020), and 9 from public restrooms is shown in Figure 3. Three sample sources, i.e., environment, human, and companion animal, within one large cluster were observed in fimH41.

**FIGURE 4:**
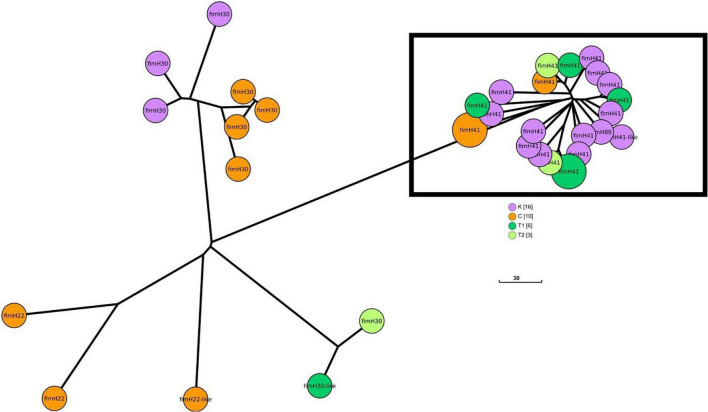
GrapeTree minimum-spanning tree based on the Enterobase cgMLST from different areas in southern Taiwan (C, Chiayi; K, Kaohsiung; T1, city center, Tainan; T2, rural area, Tainan). The largest cluster is marked with a rectangle.

Figure 4 shows the GrapeTree minimum-spanning tree based on Enterobase cgMLST from different geographic areas. The largest cluster was mainly isolated from asymptomatic individuals in Kaohsiung (K). In this cluster constituting by fimH41, in addition to Kaohsiung, other geographic sources, such as Chiayi (C), city center (T1), and rural area (T2) of Tainan, were observed within the cluster (Figure 4).

### Antibiotic-Resistant Gene Analysis by ResFinder 4.0

A further analysis of antibiotic resistance genes (Table 4) using ResFinder 4.0 from the CGE web in 22 isolates of fimH41 showed that the mean number of resistant genes was similar (human colonization: 6.58; environmental: 7.57; and dog: 8.66). All fimH41 isolates encoded the resistance genes *sitABCD* and *mdf.* There were 68% isolates harboring *blaTEM-1B*. The following three antimicrobial-resistance genes: *sul2*, *dfrA17*, and *tet(A)*, were found in approximately 50% of fimH41 isolates. Quinolone resistance-determining regions of *gyrA* mutations were found in 63.6% (*n* = 14) isolates. Except for one isolate with the D87N mutation in *gyrA*, the predominant *gyrA* mutations detected in fimH41 were S83L.

**TABLE 4:** Acquired resistance genes and associated point mutations detected in 22 isolates of ST131 *Escherichia coli* with fimH41.

Sample area	No.	*gyrA* mutation	*catA1*	*aac(3)-Iid*	*blaCTX-M-14*	*blaTEM-1B*	*aadA5*	*sul1*	*sul2*	*dfrA17*	*tet(A)*	*tet (B)*	*aph(3′′)-Ib*	*aph(6)-Id*	*mph(A)*	*Mdf*	*sitABCD*	*qacE*	Total resistant genes found
Human colonization	180	S83L	0	1	1	1	0	1	1	0	1	0	1	1	1	1	1	0	11
Human colonization	125	S83L	0	0	0	1	0	1	1	1	1	0	0	0	1	1	1	1	9
Human colonization	16	S83L	0	1	0	1	0	0	0	0	0	0	0	0	1	1	1	0	5
Human colonization	128	S83L	0	0	0	1	1	1	1	1	1	0	0	0	1	1	1	0	9
Human colonization	244	ND	1	0	0	1	0	0	1	0	0	0	1	1	0	1	1	0	7
Human colonization	354	ND	0	0	0	0	0	0	0	0	0	0	0	0	0	1	1	0	2
Human colonization	377	ND	0	0	0	0	0	0	1	0	0	1	1	1	1	1	1	0	7
Human colonization	403	S83L	0	0	0	1	1	1	1	1	1	0	1	1	1	1	1	0	11
Human colonization	700	S83L	0	1	0	1	0	0	0	0	0	0	0	0	0	1	1	0	4
Human colonization	620	ND	0	0	0	0	0	0	0	0	0	0	0	0	0	1	1	0	2
Human colonization	690	ND	0	0	0	0	0	0	0	0	0	1	0	0	0	1	1	0	3
Human colonization	515	D87N	0	0	0	0	0	0	0	0	0	0	0	0	0	1	1	0	2
Environmental (public restroom)	41	ND	1	0	0	0	0	0	0	1	0	1	0	0	0	1	1	0	5
Environmental (public restroom)	33	S83L	0	1	0	1	1	0	1	1	1	0	1	1	0	1	1	0	10
Environmental (public restroom)	59	ND	1	0	0	0	0	0	0	1	0	1	0	0	0	1	1	0	5
Environmental (public restroom)	530	S83L	0	0	0	1	1	1	0	1	1	0	1	1	1	1	1	0	10
Environmental (public restroom)	436	S83L	0	0	0	1	0	0	0	0	0	0	0	0	0	1	1	0	3
Environmental (public restroom)	600	S83L	0	0	0	1	0	0	1	1	1	0	0	0	1	1	1	0	7
Environmental (public restroom)	494	ND	1	0	0	1	0	0	1	1	0	1	1	1	0	1	1	0	9
Dog colonization	18	S83L	0	0	0	1	0	1	0	1	1	0	0	0	1	1	1	0	7
Dog colonization	2,431	S83L	0	1	0	1	0	0	1	0	1	0	1	1	0	1	1	0	8
Dog colonization	2,432	S83L	0	1	0	1	0	0	1	0	1	0	1	1	0	1	1	0	8
Total detected (%)		14 (64)	4 (18)	6 (27)	1 (5)	15 (68)	4 (18)	6 (27)	11 (50)	10 (45)	10 (45)	5 (23)	9 (41)	9 (41)	9 (41)	22 (100)	22 (100)	1 (5)	

*ND, not detected.*

## Discussion

In this survey of drug-resistant *E. coli* in public restrooms, ceftriaxone-resistant *E. coli* occurred more often in rural areas than in city centers. There were nine isolates (1.5%) found to be STc131, which was not detected in a recent public restroom survey in Minnesota, United States (Mohamed et al., 2015). However, STc131 can be found in sewage samples from a study in United Kingdom (Day et al., 2019). Geographical dominance in rural areas was not observed for the STc131 isolates. In all STc131 isolates from public restrooms and fecal colonization of human or companion animals, subtype fimH41 was predominant and circulated in southern Taiwan. Our finding indicates the fimH41 strains in the public restroom environment appear to be human origin. From the fimH type and whole-genome analysis of STc131 in our study, the possible transmission route of STc131 *E. coli* may include the following: (a) sharing of STc131 among dogs and human within a household or in the community (Johnson et al., 2009); (b) transmission through contact transmission with the contaminated surfaces and fomites at public restroom (Vardoulakis et al., 2022); and (c) the fecal-oral route transmission (Vardoulakis et al., 2022).

Some of the epidemiological findings in this study were similar to those of previous studies (Mohamed et al., 2015). We found that public parks and gas stations had a high prevalence of *E. coli*. This result is similar to that of a previous study in the United States (Mohamed et al., 2015). In addition, we obtained the greatest percentage of ceftriaxone resistant *E. coli* strains from shopping malls (5.1%) and freeway service area (5.0%) (total restrooms: 2.4%) (data not shown). In phylogenetic study, groups A (commensal-associated, 30.9%) and B2 (virulence-associated, 30.3%) were the two predominating group. This result is similar to United States study (Mohamed et al., 2015).

The ESBL-producing *E. coli* or third-generation cephalosporin-resistant *E. coli* rate in public restrooms in this study was relatively low and similar to that of the human fecal colonization rate in Taiwan (Wu et al., 2019). The character of *E. coli* in public restroom may be similar to the fecal colonization in asymptomatic adult. In addition, the ESBL-producing *E. coli* colonization rate in Taiwan is relatively low in healthy adult (<5%), which is similar to that in the Americas and Europe (Karanika et al., 2016). The cause of higher percentage of drug-resistant *E. coli* strains in rural areas is not known. We do not know if it is related to more antimicrobial use in the rural area or relative to livestock animal exposure. The percentage of multi-drug–resistant *E. coli* in the public restrooms may be related to environmental conditions, toilet design, and appropriate hygiene practices (Vardoulakis et al., 2022). This study supports the idea that bacterial surveillance in urban sewage or toilets is an economically feasible approach for continuous global surveillance and the prediction of antimicrobial resistance (Hendriksen et al., 2019).

Sequence type complex 131 was found in 1.5% of all public restrooms and 6.8% of all *E. coli*–positive sites. In our study, 77.8% of all STc131 were comprised of fimH41 in toilet samples and 81% in human fecal samples. None of nine STc131 *E. coli* (including seven of fimH41 and two of fimH30) isolates in our study produce ESBLs. We did not find STc131 with *bla*CTX-M-15 in this public restroom study. STc131 with *bla*CTX-M-15 is globally disseminated and widespread in both the community and hospitals (Louka et al., 2021). FimH41 (Clade A) is thought to have originated in Southeast Asia (Stoesser et al., 2016). Similar to previous studies, fimH41 (clades) had no mutations or had point mutations in quinolone resistance-determining regions (Johnson et al., 2013; Stoesser et al., 2016) and was associated with the O16:H5 serotype (Blanc et al., 2014; Rogers et al., 2015). Of all the *E. coli* populations in Australia and New Zealand, 8% are estimated to be comprised of ST131, whereas 4.5% are comprised of the fimH41 subclone (Rogers et al., 2015). A global collection of 700 ST131 *E. coli* showed that most isolates (80%) were from fimH30 (Clade C), whereas 4% were fimH41 (Clade A) (Ludden et al., 2020). Because most studies have focused on ESBL isolates or those from symptomatic patients, this may lead to an underestimation of fimH41 in the normal population (Zhong et al., 2015; Ludden et al., 2020). Whole-genome analysis revealed a large cluster belonging to fimH41 ST131. A recent study in Austria also reported cross-species transmission (synanthropic birds and humans) in fimH41 (Li et al., 2021). Our study supports the “one health” concept (Murphy et al., 2010; Trinh et al., 2018; Collignon and McEwen, 2019) that the same clone of fimH41 ST131 circulates among humans, dogs, and the environment. The cluster of fimH41 ST131 from different geographical areas (three counties) indicates that this strain has spread in the community in southern Taiwan.

The limitation of this study is that we only performed local surveillance of public restrooms in southern Taiwan. The collection of bacteria did not correlate with the time or effort required for regular cleanliness in each public restroom. Besides, on the sites of sampling, there, most probably, was more than one strain of *E. coli*. This may lead to underestimates of multi-drug–resistant *E. coli* and specific clonal complex. The finding of *E. coli* in restrooms may not necessarily indicate a danger to the customers. Most strains of *E. coli* detected are not pathogenic to the customer, but may indicate a lack of hygienic procedures. However, we did not correlate the findings to good or bad hygienic practices in the public restrooms in this study. In addition, we used multiplex PCR that was designed for extra-intestinal infections of humans to find the common STc in phylogenetic B2 group, and this may lead many environmental isolates classified as not-typeable. Finally, comparing the similarity in genomes of fimH41 isolated from food animals may help us understand the dissemination of fimH41 ST131 in other animal.

## Conclusion

In conclusion, our surveillance of antimicrobial-resistant *E. coli* in public restrooms showed that more drug-resistant *E. coli*, especially ceftriaxone-resistant *E. coli* occur in rural areas. ST131 was found in 1.5% of all public restrooms and most were of the fimH41 subtype. The whole-genome sequencing showed a cluster of fimH41 originating from different geographical areas and sources (human and dog colonization and restroom environment). This implies that fimH41 ST131 is in circulation among the community in southern Taiwan.

## Data Availability Statement

The datasets presented in this study can be found in online repositories. The names of the repository/repositories and accession number(s) can be found below: https://www.ncbi.nlm.nih.gov/bioproject/810247.

## Author Contributions

S-MC, J-WC, and J-LW: conceptualization. S-MC, J-WC, and JS: methodology. W-CK, and JS: validation. S-MC and J-LW: formal analysis. S-MC, C-ST, and J-LW: investigation. J-LW: writing—original draft preparation. S-MC, J-WC, and W-CK: writing—review and editing. W-CK: supervision. All authors have read and agreed to the published version of the manuscript.

## Conflict of Interest

The authors declare that the research was conducted in the absence of any commercial or financial relationships that could be construed as a potential conflict of interest.

## Publisher’s Note

All claims expressed in this article are solely those of the authors and do not necessarily represent those of their affiliated organizations, or those of the publisher, the editors and the reviewers. Any product that may be evaluated in this article, or claim that may be made by its manufacturer, is not guaranteed or endorsed by the publisher.

## Footnotes

1
https://enterobase.warwick.ac.uk/species/index/ecoli

## Abbreviations

*E. coli*: *Escherichia coli*
ESBL: extended-spectrum beta-lactamase
fimH: fimbriae gene
cgMLST: core genome multilocus sequence typing
STc: sequence type complex
SNP: single-nucleotide polymorphism
ST: sequence type.
